# Family-centered empowerment approach to optimize phosphate management among hemodialysis patients: an experimental study

**DOI:** 10.1186/s12882-023-03311-1

**Published:** 2023-09-03

**Authors:** Parvaneh Vasli, Meimanat Hosseini, Malihe Nasiri, Noushin Bakhtiari

**Affiliations:** 1 Department of Community Health Nursing, School of Nursing and Midwifery, Shahid Beheshti University of Medical Sciences, Tehran, Iran; 2 Department of Basic Sciences, School of Nursing and Midwifery, Shahid Beheshti University of Medical Sciences, Tehran, Iran

**Keywords:** Hyperphosphatemia, Empowerment, Family, Hemodialysis

## Abstract

**Background:**

This study aimed to investigate the effect of a family-centered empowerment program on hyperphosphatemia management.

**Method:**

This experimental study was performed on 80 randomly selected eligible patients with hyperphosphatemia undergoing hemodialysis. Patients were assigned randomly to two groups of family-centered empowerment program (FCEPG) and control group (CG) by coin toss (40 people per group). Data collection tools were the researcher-made Phosphate Control Knowledge Scale, the researcher-made Adherence to Dietary Restriction of Phosphorus Intake Scale, the eight-item Morisky Medication Adherence Scale, and serum phosphorus measurements. Data were collected before the intervention, one month, and three months after the intervention. Patients in FCEPG participated in a family-centered empowerment program. The statistical significance level was considered to be 0.05.

**Results:**

Inter-group comparisons showed no significant difference between FCEPG and CG in terms of the mean score of knowledge of phosphate control, adherence to dietary restriction of phosphorus intake, adherence to medication, and the mean serum phosphorus level before the empowerment program, but showed significant differences between them in these respects at one month after the program and three months after the program (*p* < 0.05). Intra-group comparisons showed a significant difference in FCEPG between the mean and standard deviation of all four variables before the empowerment program and the corresponding values one month and three months after the program (*P* < 0.05).

**Conclusion:**

The findings of this study can be used in various fields of healthcare in the hospital and community.

## Introduction

Maintenance hemodialysis is a commonly used treatment for end-stage renal disease. In this condition, phosphate becomes easily retained in the body due to decreased renal function, leading to hyperphosphatemia, a common complication in these patients [1]. For adults, hyperphosphatemia has been defined as an abnormal increase in serum phosphate level to concentrations above 5.5 mg/dl [2]. Hyperphosphatemia has a variety of consequences including the modification of vascular smooth muscle cells, which leads to vascular calcifications, hyperparathyroidism, and alterations in bone metabolism. Hyperphosphatemia has been shown to be an independent risk factor associated with increased mortality [3].

Primary interventions for hyperphosphatemia management include the dietary restriction of phosphorus intake (DRPI) and the use of phosphate binders [4, 5]. However, dialysis patients tend to have a poor understanding of DRPI [6] and find it difficult to adhere to DRPI because it requires lifestyle modification [7]. It has been estimated that roughly 43% of dialysis patients do not adhere to the prescribed dietary regimens [5]. The rate of non-adherence to phosphate binders is also quite high, as some estimates suggest that over half of dialysis patients do not follow the prescribed medication regimens [8].

There are a variety of strategies, including educational and behavioral interventions, to improve the phosphorus control of hemodialysis patients (HPs) by improving their adherence to treatment regimens and helping them engage in healthy behaviors [5, 9]. Patient empowerment interventions can potentially improve the motivation, lifestyle control, and self-confidence of HPs [10]. While healthcare workers are responsible for caring for HPs in medical settings, they need to be cared for at home by family caregivers (FCGs) and also need to take care of themselves. Thus, HPs need adequate support from FCGs who are directly involved in their care [11]. However, most FCGs have poor knowledge of the disease, symptom management solutions, and home care practices. Thus, providing education and support in the framework of family empowerment can help HPs and their FCGs take charge in managing the disease and treatment-related problems, which can reduce the complications of hemodialysis [12]. Family empowerment can help families change for the better. Family-centered empowerment makes the patients and their families more involved in health-related decision-making, thereby enabling the patients to control their health and take necessary actions to improve their health on their own [13].

Considering the importance of empowering HPs with hyperphosphatemia and their FCGs in relation to adherence to dietary and medication regimens for the purpose of controlling serum phosphorus levels, this study investigated the effect of a family-centered empowerment program (FCEP) on hyperphosphatemia management and specifically the subjects’ knowledge of phosphate control, adherence to DRPI, adherence to medication, and serum phosphorus level.

### Literature review

The effects of education programs on knowledge, adherence to dietary and medication regimens, and serum phosphorus levels of HPs with hyperphosphatemia have been the subject of several studies. In a study by Yin et al., the results showed an improvement in the HPs’ phosphate control rate, their knowledge of phosphate control, and their adherence to phosphate binder regimens after the intervention [1]. A study by Chan et al. on the effectiveness of a multidisciplinary program in controlling the hyperphosphatemia of HPs in Kuala Lumpur, Malaysia, also reported a decrease in the percentage of patients with uncontrolled phosphorus levels and an increase in the percentage of patients adhering to phosphate binder regimens [14]. In another study on the effects of a comprehensive multidisciplinary program with the goal of long-term hyperphosphatemia management, the results showed a relative improvement in serum phosphate levels as long as all parts of the program are implemented [3]. Lim et al. reported that a program they implemented to educate HPs about low phosphate diets and phosphate binders in order to control serum phosphate levels managed to improve patients’ knowledge of the appropriate time of phosphate binder consumption to some extent [15]. In a study by Stumm et al., they reported that a nursing education intervention was effective in reducing hyperphosphatemia, and that the more aware the patients were about the disease and treatment, the better their adherence was to treatment [16]. Although several studies have investigated the effect of patient education and empowerment on patients’ knowledge, dietary and medication adherence, and serum phosphate levels, this was the first study on the effect of an FCEP on hyperphosphatemia management. Given the goal of this study, which was to determine the effect of an FCEP on hyperphosphatemia management, the following hypotheses were raised and tested:

- H1: FCEP affects patients’ knowledge of phosphate control.
- H2: FCEP affects patients’ adherence to DRPI.
- H3: FCEP affects patients’ adherence to medication.
- H4: FCEP affects patients’ serum phosphorus levels.

## Methods

### Study design and setting

This study was experimental research conducted on two groups of HPs: 1- patients participating in an FCEP (hereafter refers to as FCEPG) and 2- patients in the control group (hereafter refers to as CG). All patients were recruited from the hemodialysis ward of Shahid Beheshti Hospital in Hamadan, Iran. This ward has 30 beds in three halls and provides hemodialysis to about 70 patients every day in morning and evening sessions.

Designed as a single-blind experiment, the study was arranged such that patients remain unaware of the designated interventions so that their knowledge would not affect their behavior.

### Participants and recruitment

All participants were HPs. The sample size was computed using the following formula based on the findings of Lim et al. [15], for the first type error of 0.05, the second type error of 0.10, the test power of 0.90, and the 10% sample loss. Using this method, the sample size was estimated to 40 people per group.

$$n \geq 2 \frac{\left( z_{\alpha/2} + z_{\beta}\right)^{2} \sigma^{2}}{\left(\mu_{1} - \mu_{2}\right)^{2}}$$

For sampling, the first researcher identified eligible HPs with the help of nurses. Participants of FCEPG were recruited exclusively from the morning shift and CG from the evening shift to prevent contamination. After assigning a number to each eligible HP, the numbered HPs in the morning shift were randomly assigned to FCEPG and in the evening shift to CG by casting lots. The selection of participants continued until the target sample size was reached. The two groups were matched in terms of age and gender. All participants were included in the study at the end of data collection.

Inclusion criteria were: minimum age of 35 and maximum age of 70 years, serum phosphorus level of more than 5.5 mg/dl over the last six months, presence of a first- or second-degree family member (father, mother, sibling, spouse, child, son/daughter-in-law) as the constant FCG providing care for at least six months during the dialysis session and at home, no mental illness in HP or FCG based on their report, and HP or FCG not simultaneously participating in another educational program related to diet or medication. Exclusion criteria were: HP or FCG not wanting to continue participating in the study, HP or FCG not participating in education sessions after enrollment (absence in two sessions), and deterioration of the physical condition, hospitalization, or death of HP.

### Family-centered empowerment program (FCEP)

A Family-Centered Empowerment Program (FCEP) was implemented as an intervention for FCEPG. This program was prepared clearly and simply for participants because it was assumed that some participants were probably illiterate. The content was developed based on the latest scientific sources [1, 4, 8, 17–19] and then its content validity was confirmed by two nephrologists and a nutritionist. The program was implemented in four 15–30 min sessions held for HPs with hyperphosphatemia and their FCGs during hemodialysis. Each session comprised a lecture followed by Q&A. The goals and content of these sessions and the actions taken in each session are listed in Table 1. At weeks 2, 4, 6, and 8 after the end of the fourth session, the educational content was reviewed for HPs and FCGs during hemodialysis and their questions were answered. From the beginning of the program, FCGs were asked to continuously remind HPs of the educational material and evaluate their adherence to recommendations until the final stage of data collection.

**Table 1:** FCEP implemented for HPs with hyperphosphatemia

Session	Goals	Content	Actions
1	Introduction to the researcher and research objectives Introduction to chronic kidney disease, hemodialysis, and hyperphosphatemia	- Introduction to the anatomy and physiology of the kidney - Kidney failure and its consequences - Treatment of kidney failure - Hemodialysis and its complications - Caring for vascular access and catheter site - The incidence of hyperphosphatemia in dialysis patients; optimal range for serum phosphate - Symptoms of hyperphosphatemia - Effects of hyperphosphatemia on bone, joints and other organs	Active participation in learning Q&A Review of the contents by FCGs at home
2	Learning how to control hyperphosphatemia through diet and medication	*Dietary*: - Importance of dietary adherence - Phosphorus-to-protein ratio of foods - Appropriate food choice (E.g. Consuming foods with a low phosphorus–protein ratio, but with adequate amount of protein like meat, tofu, and seafood (in a specified and controlled amount); avoidance consumption foods with a high phosphorus–protein ratio like dairy products, egg, nuts, beans, and seeds; Consuming fresh unprocessed food instead of processed food - Avoidance of phosphate additives (e.g. Consuming fresh unprocessed food instead of processed food) - Training to prepare suitable meals (e.g. soaking foods in water and boiling to reduce the dietary phosphorus content per gram of protein in foods) *Medication* - Importance of phosphate binders and their role in lowering serum phosphorus concentrations - Mode of action	Review of the contents of the previous session Q&A Active participation in learning Review of the contents by FCGs at home
3	Strengthening self-efficacy Strengthening patient’s participation in care Strengthening patient’s autonomy in care	Problem-solving method Self-efficacy The importance of autonomy and participation in self-care	Review of the contents of the previous session Q&A Active participation in learning Review of the contents by FCGs at home
4	Enhancing all aspects of empowerment through the review of previous contents, Q&A, and discussion	A summary of the contents provided	Solving a problem about controlling hyperphosphatemia that was given to HPs and FCGs in the previous session

*FCEP* Family-centered empowerment program*, HPs* hemodialysis patients, *FCGs* Family caregivers

It should be noted that participants in both CG and FCEPG also received conventional education from the hemodialysis unit. This conventional education is a short (a few minutes long) briefing about low phosphate diets that is routinely provided by a nurse or physician at discharge to HPs who have hyperphosphatemia (> 5.5 mg/dl) on the day of hemodialysis.

### Outcome measurements

#### Primary outcomes

The primary outcomes of the program were assumed to be a change in patients’ knowledge of phosphate control, adherence to DRPI, and adherence to medication, which were measured by the researcher-made Phosphate Control Knowledge Scale, researcher-made Dietary Restriction of Phosphorus Intake Scale (DRPI-S), and the eight-item Morisky Medication Adherence Scale (MMAS-8), respectively.

#### Secondary outcomes

The secondary outcome of the program was assumed to be a change in serum phosphorus level.

#### Baseline

The first researcher collected data. Baseline measurements for FCEPG and CG were performed before interventions while HPs were undergoing hemodialysis. For illiterate HPs, the questions were read aloud and answers were recorded by the first researcher.

#### Follow-ups

**Fig. 1:**
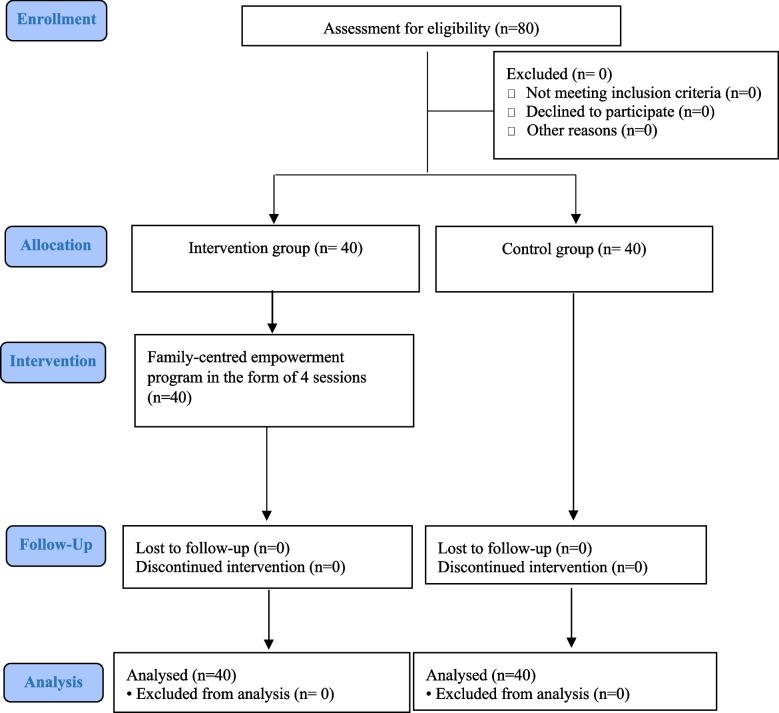
CONSORT flow chart of the study

Follow-up measurements were performed once one month after FCEP (Follow-Up1) and another time three months after FCEP (Follow-Up2) at patients’ subsequent hemodialysis appointments before the start of hemodialysis. CONSORT flow chart of the study is illustrated in Fig. 1.

### Measures

#### Sociodemographic and clinical data of participants and family caregivers

Sociodemographic and clinical information questionnaire contained 11 questions about HPs (age, gender, education level, marital status, employment status, duration of undergoing hemodialysis, primary disease, dialysis frequency, receiving vitamin D, and calcimimetics, and smoking. This questionnaire also contained seven questions about FCGs (age, gender, education level, marital status, employment status, relation to the patient, and duration of providing care to the patient).

#### Knowledge of phosphate control

Knowledge of phosphate control was measured by the Phosphate Control Knowledge Scale, which was a researcher-made tool. This questionnaire was developed by adapting the tools of similar studies and scientific recourses [14, 20, 21].

This tool has 16 items with the answers “true” (1 point), “false” (0 points), and “I don’t know” (0 points). Items 1, 9, 10, and 13 were scored in reverse. The higher the score, the greater the Knowledge of phosphate control. This scale’s minimum and maximum scores are 0 and 16, respectively (Table 2).

**Table 2:** Phosphate control knowledge scale

no	Items	True	False	I don’t know
1	The only way to control high blood phosphorus is dialysis			
2	High blood phosphorus in the body causes osteoporosis			
3	An increase in blood phosphorus can be prevented by following the diet			
4	Failure to control high levels of blood phosphorus can lead to heart problems			
5	Legumes, milk, and nuts have high amounts of phosphorus			
6	Timely and correct use of phosphorus-reducing drugs prevents the increase of blood phosphorus levels			
7	Constipation, nausea, and vomiting are common side effects of phosphorus-reducing drugs			
8	In the case of the interaction of phosphorus-reducing drugs with other drugs, there is a possibility of reducing the absorption of consumed drugs			
9	Fresh beef, rice, milk, or almond milk contains low amounts of phosphorus			
10	When the amount of phosphorus in the blood is more than 4.5 mg, its effects are revealed in the body			
11	Carbonated drinks, dairy products, and meat contain high amounts of phosphorus			
12	Itching and redness of the eyes are symptoms of increased blood phosphorus			
13	Blood phosphorus levels can be controlled only with medication in people undergoing dialysis			
14	Osteoporosis is the most common cause of bone fractures in dialysis patients with high blood phosphorus			
15	The most common cause of increased phosphorus in the body is kidney failure			
16	By performing a 24-h blood and urine test, it is possible to determine the amount of phosphorus in the blood			

To check the validity of the Phosphate Control Knowledge Scale, it was submitted for review to 10 faculty members of Shahid Beheshti University of Medical Sciences. During this process, in addition to qualitative content validity assessment, the necessity of the questions and their relevance to the research objectives were also evaluated based on Content Validity Ratio (CVR) and Content Validity Index (CVI), respectively [22]. Based on the Lawshe table, all items earned CVR scores of more than 0.62, which confirmed their necessity [23]. The Scale-level Content Validity Index (S-CVI) was calculated to 0.93. The face validity of the scale was verified by asking 10 HPs with hyperphosphatemia to evaluate it in terms of comprehensibility, transparency, and fluency.

The reliability of this tool was established by test–retest and intra-class correlation calculation based on the responses of 10 participants who filled out the questionnaire two times with a 10-day interval. Using this method, the intra-class correlation coefficient was calculated to 0.75. The Cronbach’s alpha of the tool based on the responses of 20 HPs who were not included in the study was calculated to 0.88 that showed the instrument was reliable [22].

#### Adherence to dietary restriction of phosphorus intake

Adherence to DRPI was measured by the researcher-made Dietary Restriction of Phosphorus Intake Scale (DRPI-S), which was also developed by the researchers based on scientific sources [18, 19]. This scale has 10 items, each with five responses scored on a 5-point Likert scale: “Never” (1 point), “Rarely” (2 points), “Sometimes” (3 points), “Most of the time” (4 points) and “Always” (5 points). Items 8 and 9 were scored in reverse. The minimum and maximum scores of this scale are 10 and 50, respectively. The higher the score, the greater the adherence to dietary restriction of phosphorus intake (Table 3).

**Table 3:** Dietary restriction of phosphorus intake scale

no	Items	Always	Most of the time	Sometimes	Rarely	Never
1	In my diet, I pay more attention to the taste than the amount of phosphorus in them					
2	I use foods low in phosphorus, such as bread, beef, peas, green beans, and drink combinations without phosphorus					
3	I get the most information about phosphorus in food from a doctor, nurse, or nutritionist					
4	My goal in following the recommended diet is to minimize waste products in the blood					
5	I only change my diet after consulting a doctor or nutritionist					
6	I ask the doctor, nurse, or nutritionist about the reasons for following the diet					
7	I try to have variety in my diet within the recommended range					
8	During the last two weeks, I did not follow the recommended diet					
9	I don't follow my diet at work, traveling, or partying					
10	I avoid consuming foods with high phosphorus, such as dairy products, fish, nuts, peas, beans, lentils, and soybeans					

The validity and reliability of this tool were established in the same way as described for the Phosphate Control Knowledge Scale. All items of this tool earned CVR and CVI scores of more than 0.62 and 0.96, respectively, which confirmed their necessity and relevance. The tool’s intra-class correlation coefficient and Cronbach’s alpha were determined to be 0.91 and 0.83, respectively, which confirmed the reliability of the tool [22].

#### Adherence to medication

Adherence to medication was measured using the version of eight-item Morisky Medication Adherence Scale (MMAS-8)^1^ [24]. US Copyright laws protect the use of the MMAS-8, and an agreement for use is required. The authors have obtained a license from the scale inventor Donald E. Morisky (Certificate number: 7668–6979-6950–5104-2081). This self-report instrument has been used in several studies to assess adherence to medication in chronic diseases. MMAS-8 is a highly credible tool for measuring patients’ adherence to their medication [25]. This tool is low-cost, noninvasive, with minimal burden on a participant, easy to administer, and offers flexible timing. It has been validated in many countries and translated into several languages [26]. The first seven items having a “Yes” (score 0), and “No” (score 1) responses, except question 5, which reverses the score. For item 8, a patient can choose an answer on a 5-point Likert scale as “Never/Rarely” (score 1), “Once in a while” (score 0.75), “Sometimes” (score 0.5), “Usually” (score 0.25), “All the time” (score 0), expressing how often happens that a patient does not take his medications. MMAS-8 scores can range from zero to 8 points. Patients who scored 8 points, < 8 to > 6 points and ≤ 6 points on the scale were considered to have high, medium and low adherence, respectively [27, 28]. The Cronbach’s alpha of MMAS-8 based on the responses of 20 HPs who were not included in the study was calculated to 0.69.

#### Serum phosphorus measurement

At the dialysis ward, serum phosphorus level measurement was a routine procedure for all HPs with hyperphosphatemia while fasting. This procedure involved a nurse taking a blood sample, sending it to the hospital’s laboratory, and then recording the results in the HP’s medical file at 8 AM. The first researcher (responsible for implementing the interventions and collecting the data) extracted the participating HPs’ serum phosphorus level data from their medical files and recorded them in the data collection sheets.

The blood samples of the CG participants undergoing evening hemodialysis sessions were also prepared in the morning. CG participants were asked to attend the hemodialysis department at 8 AM for the data collection sessions (baseline, follow-up 1, and follow-up 2) in a fasting state for blood sampling. The researcher went to their homes to take the blood sample for participants who did not want to go to the hemodialysis department in the morning.

### Data analysis

The collected data were processed using the software SPSS version 26. The data were analyzed by descriptive statistical methods such as computing numerical measures (mean and standard deviation) and inferential tests. The independent t-test and the chi-square test were used to compare FCEPG and CG in terms of demographic variables. The independent t-test was also used to compare FCEPG and CG in terms of knowledge of phosphate control, adherence to DRPI, adherence to medication, and serum phosphorus level. The GLM-repeated measures ANOVA was used to make intra-group comparisons between the three measurement stages: Baseline, Follow-Up1 (one month after FCEP), and Follow-Up2 (three months after FCEP). For all tests and analyses, the significance level was considered to be 0.05.

## Results

The mean age of HPs in FCEPG and CG was 48.16 ± 16.6 and 46.35 ± 14.86, respectively. Participants in FCEPG and CG were undergoing hemodialysis for respectively 8.63 ± 4.09 and 7.65 ± 4.17 years on average. There was no significant difference between the participants in FCEPG and CG in terms of any of the variables.

Also, the mean age of FCGs in FCEPG and CG was 38.28 ± 10.23 and 39.65 ± 10.89, respectively. There was no significant difference between the two groups in terms of the age of HPs or FCGs.

FCGs of the two groups were providing care to HPs for 9.82 ± 3.63 and 9.13 ± 3.42 years, respectively. Other demographic information of the participants is given in Table 4.

**Table 4:** Baseline sociodemographic and clinical data of participants and their FCGs in FCEPG and CG

**Group** **Variable**	**FCEPG**		**CG**		*P*-value^a^
	n	%	n	%	
**Patients**					
**Gender**					
Female	14	35	16	40	0.64
Male	26	65	24	60	
**Marital status**					
Single	10	25	10	25	0.19
Married	5	12.5	12	30	
Widow/ Divorced	25	62.5	18	45	
**Education level**					
Illiterate/Primary school	11	27.5	10	25	0.08
Middle school	6	15	9	22.5	
High school /diploma	8	20	15	37.5	
University degree	15	37.5	6	15	
**Employment status**					
Homemaker	5	12.5	5	12.5	0.78
Unemployed	5	12.5	6	15	
Self-employed	6	15	4	10	
Employee	17	42.5	13	32.5	
Retired	7	17.5	12	30	
**Primary diseases**					
Diabetic nephropathy	14	35	11	27.5	0.39
Hypertensive nephropathy	9	22.5	12	30	
Chronic glomerulonephritis	6	15	7	17.5	
Immune nephropathy	7	17.5	5	12.5	
Urinary tract infections	4	10	3	7.5	
Other diseases	0	0	2	5	
**Dialysis frequency**					
2 times/ week	8	20	10	25	0.25
3 times/ week	32	80	30	75	
**Receiving vitamin D**					
Yes	28	70	26	65	0.68^a^
No	12	30	14	35	
**Receiving phosphorus adsorbents**					
Calcium carbonate	26	65	29	72.5	0.86
Calcium citrate	12	30	11	27.5	
Others	2	5	0	0	
**Smoking**					
Yes	20	50	17	42.5	0.56
No	20	50	23	57.5	
**Family caregivers**					
**Gender**					
Female	21	52.5	22	55	0.82^a^
Male	19	47.5	18	45	
**Marital status**					
Single	9	22.5	11	27.5	0.81^a^
Married	12	30	13	32.5	
Widow/ Divorced	29	44.5	16	40	
**Education level**					
Illiterate/ Primary school	11	27.5	17	42.5	0.14^a^
Middle school	9	22.5	12	30	
High school/ diploma	7	17.5	6	15	
University degree	13	32.5	5	12.5	
**Employment status**					
Homemaker	12	30	8	20	0.39^a^
Unemployed	6	15	6	15	
Self-employed	6	27.5	8	20	
Employee	11	27.5	10	25	
Retired	5	12.5	8	20	
**Relationship with the patient**					0.91^a^
Mother	5	12.5	3	7.5	
Father	7	17.5	6	15	
Spouse	6	15	7	17.5	
Child	7	17.5	8	20	
Sister/ brother	3	7.5	1	2.5	
daughter-in-law/ son-in-law	12	30	15	37.5	

*FCGs* Family caregivers*, FCEPG* Family-centered empowerment program group*, CG* Control group

^a^Chi-square test

The results showed no significant difference between FCEPG and CG in terms of the mean and standard deviation of knowledge of phosphate control, adherence to DRPI, and adherence to medication (primary outcomes) at Baseline, but showed significant differences between them in these respects at Follow-Up1 and Follow-Up2 (*P* < 0.05), indicating a change in the scores of all four variables after the implementation of FCEP (Table 5).

**Table 5:** Comparison changes of the variables in FCEPG and CG

Group Variables		FCEPG	CG	*P*-value
Knowledge of phosphate control	Baseline	4.35 ± 2.31	5.20 ± 2.09	0.08^a^
	Follow-up1	9.32 ± 2.05	6.15 ± 2.33	0.001^a^
	Follow-up2	10.37 ± 2.52	7.52 ± 2.57	0.001^a^
	Follow-ups vs. Baseline	0.001^b^	0.001^b^	
Adherence to DRPI	Baseline	24.15 ± 7.13	26.13 ± 6.46	0.19^a^
	Follow-up1	35.75 ± 7.26	29.48 ± 6.96	0.001^a^
	Follow-up2	37.10 ± 7.10	31.25 ± 7.06	0.001^a^
	Follow-ups vs. Baseline	0.001^b^	0.001^b^	
Adherence to medication	Baseline	5.75 ± 0.5	5.5 ± 0.75	0.18^a^
	Follow-up1	7.25 ± 1.25	5.75 ± 1.25	0.001^a^
	Follow-up2	8 ± 0.75	6.25 ± 0.5	0.001^a^
	Follow-ups vs. Baseline	0.001^b^	0.001^b^	
Serum phosphorus level	Baseline	6.57 ± 0.98	6.75 ± 1.7	0.72^a^
	Follow-up1	6.65 ± 2.02	6.1 ± 1.28	0.005^a^
	Follow-up2	5.87 ± 1.65	6.7 ± 1.24	0.005^a^
	Follow-ups vs. Baseline	0.005^b^	0.005^b^	

Data are represented as mean ± standard deviations

*FCEPG* Family-centered empowerment program group*, CG* Control group*, DRPI* Dietary restriction of phosphorus intake

^a^*p*-values for comparing scores between the FCEPG and CEG, at baseline (derived from independent t-tests)

^b^*p*-value for comparing differences between follow-ups and baseline (derived from GLM-repeated measure ANOVA)

Intra-group comparisons between Baseline, Follow-Up1, and Follow-Up2 showed significant changes in the mean and standard deviation of knowledge of phosphate control, adherence to DRPI, and adherence to medication in both FCEPG and CG, but this change was more pronounced in FCEPG than in CG (Table 5). The rising scores in CG may be related to the conventional hemodialysis education provided to HPs with hyperphosphatemia. As the results of Table 5 demonstrate, in FCEPG, the mean scores of all three variables increased significantly from Baseline to Follow-Up1, from Follow-Up1 to Follow-Up2, and from Baseline to Follow-Up2.

Considering the significant differences between FCEPG and CG in terms of the mean scores of knowledge of phosphate control, adherence to DRPI, adherence to medication at Follow-Up1 and Follow-Up2, and the more romanced increase in the mean scores of FCEPG compared to CG, it can be concluded that the implemented FCEP managed to affect knowledge of phosphate control, adherence to DRPI, adherence to medication, which confirms the hypotheses H1, H2, and H3.

As shown in Table 5, while there was no significant difference between FCEPG and CG in terms of serum phosphorus level (secondary outcome) at Baseline, there was such a difference between the two groups at Follow-Up1 and Follow-Up2 (*P* < 0.05). In both FCEPG and CG, serum phosphorus levels also significantly changed over time (*P* < 0.05). The point to note is that in FCEPG, serum phosphorus levels slightly increased at Follow-Up1 and then sharply dropped at Follow-Up2. According to Table 6, the increase in FCEPG serum phosphorus level from Baseline to Follow-Up1 was insignificant, but the decrease in serum phosphorus level from Baseline to Follow-Up2 and from Follow-Up1 to Follow-Up2 was significant (*P* < 0.05). The reason for the initial increase in serum phosphorus level at Follow-Up1 could be that the intervention has not been completely effective until week 4 after FCEP, but the level of serum phosphorus has decreased with the repeated review of the educational content in weeks 6 and 8 after FCEP. From the above results, it can be cautiously concluded that the implemented FCEP can been able to decrease the patients’ serum phosphorus level.

**Table 6:** Pairwise comparisons of changes of the variables in FCEPG

Variables		Mean difference	Standard error	*P*-value^a^
Knowledge of phosphate control	Follow-up1 vs. Baseline	-4.85	1.1843	0.001
	Follow-up2 vs. Baseline	-6.12	1.1843	0.001
	Follow-up2 vs. Follow-up1	-1.05	1.1843	0.001
Adherence to DRPI	Follow-up1 vs. Baseline	-11.60	0.286	0.001
	Follow-up2 vs. Baseline	-12.95	0.3016	0.001
	Follow-up2 vs. Follow-up1	-1.35	0.1845	0.001
Adherence to medication	Follow-up1 vs. Baseline	-1.5	0.131	0.001
	Follow-up2 vs. Baseline	-2.25	0.190	0.001
	Follow-up2 vs. Follow-up1	-0.75	0.167	0.001
Serum phosphorus level	Follow-up1 vs. Baseline	-0.09	0.3407	0.936
	Follow-up2 vs. Baseline	0.71	0.3407	0.001
	Follow-up2 vs. Follow-up1	0.78	0.3407	0.001

Data are represented as mean ± standard deviations

*FCEPG* Family-centered empowerment program group*, DRPI* Dietary restriction of phosphorus intake

^a^*p*-values for comparing differences between follow-up1 and baseline, follow-up2 and baseline, and follow-up2 and follow-up1 (derived from pairwise comparisons Bonferroni test)

## Discussion

The goal of this study was to investigate the effect of an FCEP on hyperphosphatemia management in HPs. The results showed that FCEP could have a positive impact on the patients’ knowledge of phosphate control, adherence to DRPI, and adherence to medication as primary outcomes and on their serum phosphorus levels as the secondary outcome.

Regarding the first research hypothesis, namely the effect of FCEP on knowledge of phosphate control, although we could not find any study on the effect of family-centered education on HPs’ knowledge, we found several studies showing that education and empowerment can improve the knowledge of patients with hyperphosphatemia. The study of Yin et al. for example showed that an intensive education program focused on phosphate control can improve HPs' phosphate control knowledge scores [1]. Hjemås et al. showed that education can increase HPs’ knowledge of the need to control hyperphosphatemia and also their knowledge of how to control it through the use of phosphate binders [20]. A systematic review also reported that educational and behavioral interventions could improve HPs’ knowledge of phosphate level control [29]. According to these results, education and empowerment appear to be an effective approach for improving the knowledge and awareness of patients with hyperphosphatemia and perhaps controlling their serum phosphate levels [30].

In relation to the second hypothesis of this study, namely the effect of FCEP on adherence to DRPI, the literature contains several studies with findings similar to ours. In one of these studies, Montazami et al. reported that home visit-based FCEP can be an effective way to improve adherence to dietary and medication regimens in patients with acute coronary syndrome [31]. Asgari et al. also reported that their FCEP managed to improve HPs’ self-care capability [32]. In a study conducted in Egypt, it was found that empowering HPs and their FCGs helped them manage their health-related problems and improved their self-efficacy [12]. In a study conducted in Australia, the results showed that education programs based on mobile texting can improve HPs’ adherence to dietary regimens [33].

Regarding the third hypothesis of the study, namely the effect of FCEP on adherence to medication, one study has shown that family-centered education is more effective than patient-centered education in improving HPs’ adherence to treatment and especially medication regimens [34]. According to a systematic review and meta-analysis, educational interventions tend to have positive effects on patients’ adherence to dietary regimens and treatment [9]. In a meta-analysis study, it was concluded that educational interventions could significantly change patients’ adherence to treatment [35]. In the study of Yin et al., the results showed that the implementation of an intensive education program focusing on phosphate control could improve HPs’ adherence to medication and consumption of phosphate binders [1]. The results of a quasi-experimental study also showed that education could improve HPs’ adherence to medication [36].

Concerning the fourth hypothesis of the study, namely the effect of FCEP on the secondary outcome, i.e. serum phosphorus level, the results of a study by Rabiei showed that FCEP could reduce the negative outcomes of CKD in patients [34]. The results of Bahramnezhad et al. showed that family-centered education is more effective than patient-centered education in reducing the complications of hemodialysis [37]. The studies of Yin et al. and Lim et al. both showed that an educational program focusing on phosphate control could increase the percentage of HPs with normal serum phosphorus levels [1, 15]. Chan et al. reported that a multidisciplinary program aimed at controlling hyperphosphatemia in HPs could be highly effective in reducing the percentage of patients with uncontrolled phosphorus levels [14]. In a study by Brauer et al., the results indicated that patients’ serum phosphorus levels can be controlled by holding education and discussion sessions about diet and phosphate binders [3]. In a meta-analysis of the literature on the subject, it was concluded that psychosocial and educational interventions can be effective in improving HPs’ adherence to treatment and control of dialysis-related factors, including serum phosphorus level [35].

The present study is significant from these two perspectives. First, it focuses on the empowerment of not only HPs but also FCGs as people who play an essential role in the health promotion of these patients. Second, in addition to exploring the effect of FCEP on primary outcomes such as knowledge of phosphate control, adherence to DRPI, and adherence to medication, this study examines the effect of FCEP on the most important outcome for patients with hyperphosphatemia, i.e. serum phosphorus level.

## Conclusion

This study aimed to determine the effect of FCEP on hyperphosphatemia management in HPs. The results showed that in addition to affecting knowledge of phosphate control, adherence to DRPI, and adherence to medication as primary outcomes, FCEP could improve the serum phosphorus level of patients with hyperphosphatemia as the secondary outcome.

Considering the effect of FCEP on patients with hyperphosphatemia in terms of primary and secondary outcomes, the authors recommend the implantation of this program by healthcare workers in hospital hemodialysis wards as well as home-visiting to improve the health of hemodialysis patients with hyperphosphatemia. It is also recommended to incorporate FCEP into the continuing education programs developed for healthcare workers and the care quality evaluations as a step toward reducing the potential implications of hyperphosphatemia such as frequent hospitalization and health deterioration for this group of patients.

The authors believe that further research must still be conducted on the impact of family-centered empowerment programs focusing on home care as well as distance education on patients’ adherence to treatment and control of hyperphosphatemia. The most important limitation of this study was the poor cooperation and impatience of some participants due to the concurrence of FCEP and administration of research tools with their hemodialysis procedure, which may have affected the results. Although this study was performed in one hospital in Iran, the authors believe that the findings can be generalized to the entire country and even other countries.

## Data Availability

The data used to support the findings of this study are available from the corresponding author upon reasonable request.

## Footnotes

1 The MMAS-8 Scale, content, name, and trademarks are protected by US copyright and trademark laws. Permission for use of the scale and its coding is required. A license agreement is available from MMAR, LLC., www.moriskyscale.com.
